# Longevity cosmeceuticals as the next frontier in cosmetic innovation: a scientific framework for substantiating product claims

**DOI:** 10.3389/fragi.2025.1586999

**Published:** 2025-05-22

**Authors:** Wannita Klinngam, Athit Chaiwichien, Supawadee Osotprasit, Uracha Ruktanonchai, Mayuree Kanlayavattanakul, Nattaya Lourith, Amaraporn Wongrakpanich, Veerawat Teeranachaideekul, Tawin Iempridee

**Affiliations:** ^1^ National Nanotechnology (NANOTEC), National Science and Technology Development Agency, Pathum Thani, Thailand; ^2^ School of Cosmetic Science, Mae Fah Luang University, Chiang Rai, Thailand; ^3^ Phytocosmetics and Cosmeceuticals Research Group, School of Cosmetic Science, Mae Fah Luang University, Chiang Rai, Thailand; ^4^ Department of Pharmacy, Faculty of Pharmacy, Mahidol University, Bangkok, Thailand

**Keywords:** longevity cosmeceuticals, aging hallmarks, geroprotectors, skinspan, skin aging biomarkers

## Abstract

The field of anti-aging research has made remarkable strides with the identification of geroprotectors—compounds capable of extending healthspan and lifespan in animal models—presenting promising implications for human longevity. Building on these advances, we propose a novel product category: longevity cosmeceutical actives and products. Unlike conventional anti-aging products that primarily target superficial signs of aging, longevity cosmeceuticals address the molecular hallmarks of aging, fundamentally enhancing skin health and longevity. To clearly distinguish these scientifically validated products from marketing-driven claims, we define, for the first time, longevity cosmeceutical actives and products based on stringent criteria: (1) they must directly target and modulate established hallmarks of skin aging; (2) they must demonstrably extend “skinspan” over time, reflected by improved skin viability, structure, and functional integrity; and (3) their efficacy must be validated through clinical trials, preferably with post-trial skin biopsies to evaluate aging hallmark biomarkers, along with comprehensive safety assessments. This review explores molecular hallmarks of skin aging, highlights geroprotective compounds with potential cosmeceutical applications, and recommends essential biomarkers for assessing prevention of rapid biological aging. Additionally, we propose methodologies for skinspan assessment and emphasize the importance of robust clinical trial designs. By establishing these scientifically rigorous standards, we aim to drive innovation, substantiate longevity claims, and transform the cosmetic industry toward meaningful biological improvements in skin health.

## 1 Introduction

**FIGURE 1:**
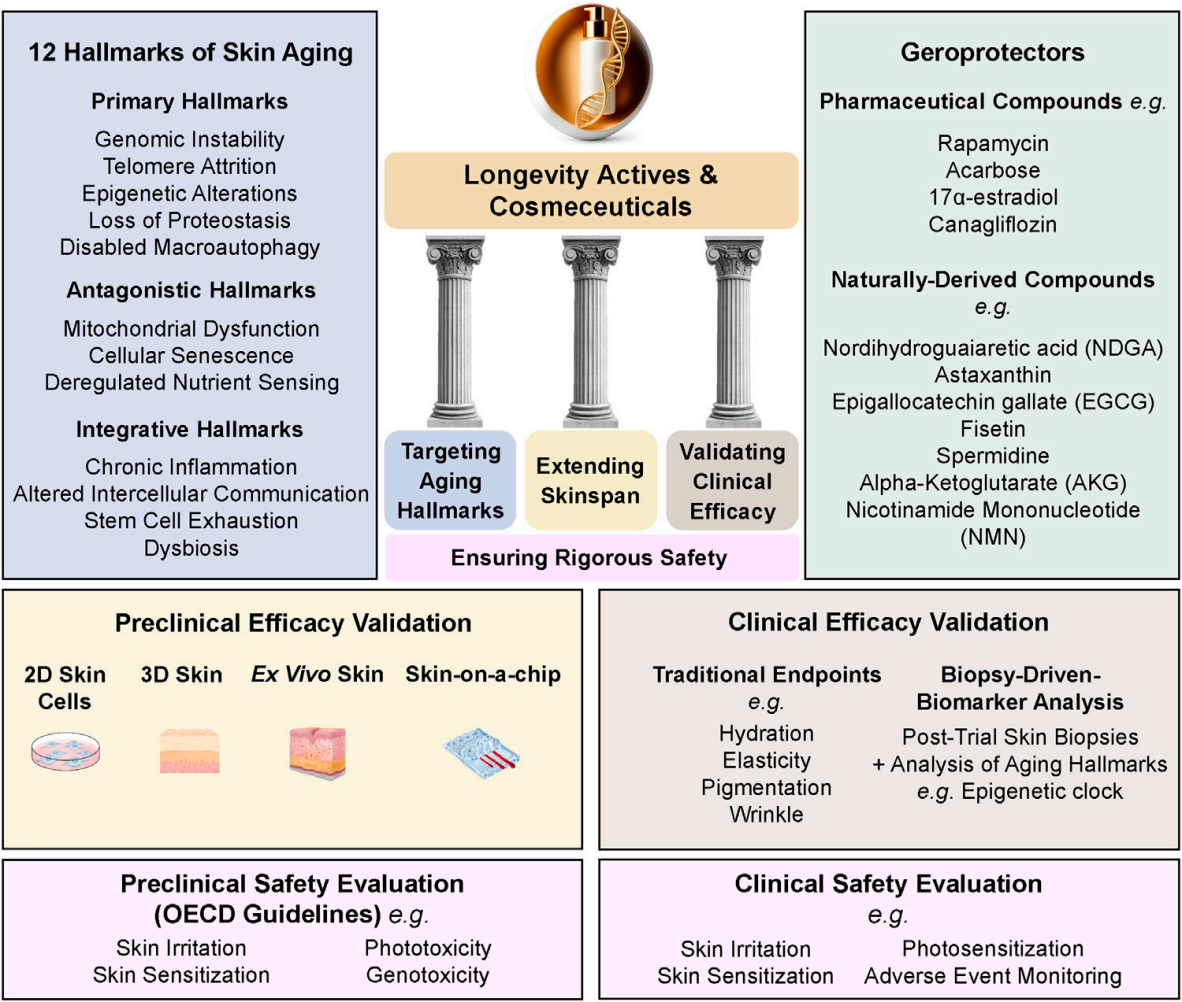
Conceptual Framework for Longevity Cosmeceuticals. The framework outlines four essential components: Targeting Aging Hallmarks, Extending Skinspan, Validating Clinical Efficacy, and Ensuring Rigorous Safety. It integrates the 12 Hallmarks of Skin Aging, representative geroprotective compounds (naturally derived and pharmaceutical), advanced preclinical evaluation models (2D/3D cultures, *ex vivo* skin, skin-on-a-chip), and clinical validation approaches, including traditional endpoints, aging biomarker analyses, and safety assessments conducted through both preclinical (OECD guidelines) and clinical evaluations.

As the aging population grows, consumer demand is shifting toward skincare solutions that go beyond traditional cosmetics, seeking products that target the biological mechanisms of aging rather than merely alleviating its signs. Although the term “longevity cosmeceuticals” has begun to appear in industry and marketing narratives, it remains undefined and unsupported by peer-reviewed scientific literature. In this manuscript, we define longevity cosmeceutical actives and products as skincare innovations grounded in advances in aging hallmarks and geroscience, offering a transformative approach to promoting and preserving skin health. This shift is reflected in the 2024 Cosmetic 360 exhibition, which highlights longevity-driven innovations in regenerative skincare, cellular anti-senescence, and biomolecular longevity strategies, emphasizing proteomics, genomics, epigenetics, and sustainability to enhance both cutaneous health and product durability (COSMETIC360, 2024). Unlike conventional products driven by marketing claims, longevity cosmeceuticals require scientific validation, bridging dermatology and longevity science. This review examines the hallmarks of skin aging, geroprotectors, biomarkers, skinspan, and clinical testing, establishing a rigorous scientific framework for this rapidly evolving category. To provide an integrated overview of these concepts, the conceptual framework for longevity cosmeceuticals is presented in Figure 1.

### 1.1 Hallmarks of aging and their relevance to skin aging

Aging is a progressive process characterized by cellular decline—manifested as reduced cell functionality, accumulation of senescent cells, depletion of regenerative stem cell pools, and molecular damage—alongside tissue and organ deterioration, dysfunction, and an increased risk of chronic diseases, including cardiovascular disease, diabetes, arthritis, cancer, neurodegenerative disorders, and other metabolic conditions (Guralnik et al., 2020a; Guo et al., 2022). Skin aging is a multifaceted process influenced by both intrinsic and extrinsic factors, leading to structural and physiological changes in the skin. Intrinsic aging is primarily driven by endogenous oxidative stress and accumulated cellular damage, whereas extrinsic aging is induced by environmental factors, such as ultraviolet (UV) radiation and pollution, which accelerate skin deterioration (Shin et al., 2023). In addition to intrinsic and extrinsic aging mechanisms, cumulative psychosocial and environmental stress across the life course has been associated with accelerated biological aging (Suglia et al., 2024).

**FIGURE 2:**
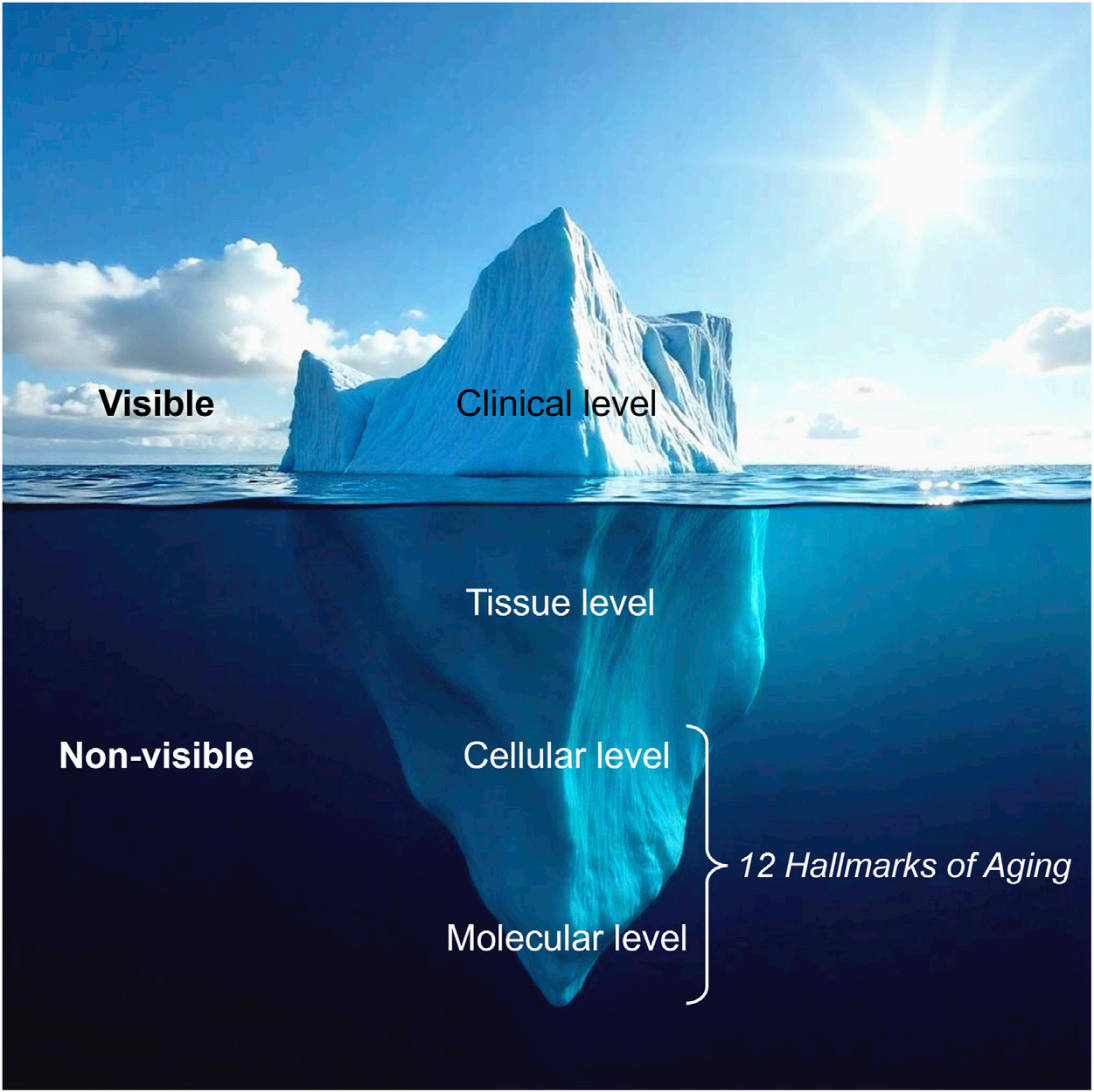
The iceberg model of skin aging. Visible signs of aging represent only the surface level, while deeper, non-visible biological processes at the tissue, cellular, and molecular levels drive skin deterioration. The twelve hallmarks of aging primarily affect the cellular and molecular levels, underlying the fundamental mechanisms of skin aging (Modified from (QIMA-LifeSciences, 2025)).

Skin aging is most visibly recognized through wrinkles, loss of elasticity, and pigmentation; however, these external changes are merely surface reflections of deeper biological dysfunctions. The iceberg model of skin aging (Figure 2) illustrates this relationship, where clinical signs (visible aging) are driven by underlying, non-visible mechanisms occurring from the molecular to tissue levels. At its core, aging is governed by twelve interconnected biological processes known as the twelve hallmarks of aging, which primarily originate at the molecular and cellular levels before manifesting structurally in the skin (López-Otín et al., 2023).

The twelve hallmarks of aging, classified as primary (damage drivers), antagonistic (initially protective but later harmful), and integrative (systemic frailty contributors), provide a framework for understanding molecular aging and developing intervention strategies (López-Otín et al., 2023; Tartiere et al., 2024). Given skin’s continuous renewal demands and exposure to environmental stressors, the skin is particularly vulnerable to aging processes, leading to barrier dysfunction, extracellular matrix degradation, and impaired regeneration (Wong and Chew, 2021; Jin et al., 2023). Understanding these hallmarks is critical for developing longevity-focused interventions that do more than mask aging signs—they aim to modulate the biological pathways driving skin aging, ultimately preserving skin health and extending skin longevity.

#### 1.1.1 Primary hallmarks: Initiating aging-related damage

**Genomic instability** from UV exposure, pollution, and oxidative stress leads to mutations, chromosomal aberrations, and impaired repair mechanisms, driving premature senescence and increased skin cancer risk (Vijg and Dong, 2020; Usategui et al., 2022; Jin et al., 2023). **Telomere attrition** limits stem cell proliferation and tissue regeneration, resulting in thinning epidermis, delayed wound healing, and reduced elasticity (Martinez et al., 2014; Srinivas et al., 2020; Jacczak et al., 2021). **Epigenetic alterations**, including DNA methylation and histone modifications, disrupt gene regulation, barrier function, and pigmentation, impairing skin renewal (Frye et al., 2007; Driskell et al., 2012; Li et al., 2017). **Loss of proteostasis** leads to misfolded protein accumulation, weakening extracellular matrix (ECM) integrity, collagen structure, and stress resistance, promoting wrinkles and barrier dysfunction (Bulteau et al., 2007). **Disabled macroautophagy**, essential for cellular waste removal, worsens oxidative stress and inflammation, accelerating ECM degradation and loss of elasticity (Kohl et al., 2011; Kim et al., 2018).

#### 1.1.2 Antagonistic hallmarks: initially protective, later detrimental

**Mitochondrial dysfunction** impairs ATP production, increasing ROS damage, which weakens fibroblast function, collagen integrity, and barrier resilience, accelerating photoaging (Hamanaka et al., 2013; Quan et al., 2015; Nakashima et al., 2017; Lan et al., 2019; Oblong et al., 2020). **Cellular senescence,** driven by stress-induced growth arrest, results in senescence-associated secretory phenotype (SASP) secretion, promoting chronic inflammation, ECM disruption, and delayed healing, leading to wrinkle formation and skin thinning (Blume-Peytavi et al., 2016; Ho and Dreesen, 2021; Danczak-Pazdrowska et al., 2023). **Deregulated nutrient sensing**, involving mTOR, AMPK, and IGF-1, disrupts metabolic homeostasis and repair mechanisms, where mTOR hyperactivation accelerates collagen degradation, and reduced AMPK/SIRT1 impairs skin resilience (Zouboulis and Makrantonaki, 2011; Choi et al., 2016; Golubtsova et al., 2017; Chung et al., 2019; Bielach-Bazyluk et al., 2021).

#### 1.1.3 Integrative hallmarks: driving systemic frailty

**Chronic inflammation (“inflammaging”)**, fueled by senescent cells and immune dysregulation, leads to barrier dysfunction, prolonged wound healing, and increased sensitivity to environmental damage (Quan et al., 2011; Meyer et al., 2017). **Altered intercellular communication**, marked by Notch, Wnt, and IGF-1 signaling decline, weakens epidermal differentiation, collagen production, and tissue homeostasis, accelerating structural degradation (López-Otín et al., 2023). **Stem cell exhaustion** reduces keratinocyte and fibroblast renewal, causing thinner, fragile skin with diminished self-repair capacity (Liu et al., 2019). **Dysbiosis**, or skin microbiome imbalance, disrupts immune homeostasis and barrier integrity, increasing susceptibility to inflammation, infections, and premature aging (Howard et al., 2022; Ratanapokasatit et al., 2022).

### 1.2 Geroprotectors: targeting aging mechanisms

Geroprotectors extend lifespan by delaying aging-related decline and improving biomarkers with minimal side effects (Moskalev et al., 2017; Moskalev, 2023). The Interventions Testing Program (ITP), initiated by the National Institute on Aging (NIA), has identified key pharmaceutical geroprotectors with significant lifespan extension in mice. **Rapamycin**, an mTOR inhibitor, extends lifespan by 14%–26% in females and 9%–23% in males through autophagy enhancement and metabolic regulation (Nadon et al., 2017). **Acarbose**, an α-glucosidase inhibitor, increases male lifespan by 22% and female lifespan by 5% by slowing glucose absorption and reducing metabolic stress (Harrison et al., 2014). **17α-estradiol**, with anti-inflammatory and antioxidant properties, extends male lifespan by 12% but has little effect on females (Harrison et al., 2014). **Canagliflozin**, an SGLT2 inhibitor that mimics caloric restriction by inhibiting renal glucose reabsorption, increases male lifespan by 14%. (Miller et al., 2020). While effective in aging models, their pharmaceutical nature limits direct cosmeceutical applications.

Naturally derived geroprotectors hold greater potential for longevity-focused cosmeceuticals compared to clinical drugs due to their broader applicability and ability to modulate aging pathways. **Nordihydroguaiaretic acid (NDGA)**, an autophagy activator and p300 inhibitor, extends male lifespan by 8%–10% and enhances skin barrier function in atopic dermatitis models (Kim et al., 2012; Harrison et al., 2014; Tezil et al., 2019). **Astaxanthin**, a Nrf2 activator and mitochondrial protectant, extends male lifespan by 12%, while improving elasticity, hydration, and wrinkle reduction by mitigating oxidative damage (Davinelli et al., 2018; Harrison et al., 2024). **Epigallocatechin gallate (EGCG)**, a green tea polyphenol, increases lifespan by 7% in females and 10% in males via AMPK activation and fibroblast protection, enhancing skin hydration, elasticity, and pigmentation balance, particularly in photoaged skin (Jiang et al., 2024; Sun et al., 2024).

Several natural geroprotectors, though not validated by the Interventions Testing Program (ITP), have demonstrated significant lifespan extension and skin benefits in independent studies. **Fisetin**, a senolytic flavonoid, extends median and maximum lifespan by eliminating senescent cells, reducing inflammation, and strengthening the skin barrier (Yousefzadeh et al., 2018; Takaya et al., 2024). Topically, it reduces UVB-induced wrinkles, enhances collagen levels, and inhibits MMPs and COX-2 (Wu et al., 2017). **Spermidine**, an autophagy-boosting polyamine, extends lifespan by 15%–30%, improves skin hydration, elasticity, wound healing, and preserves collagen integrity (Eisenberg et al., 2009; Kim et al., 2021). **GlyNAC**, a combination of **Glycine (Gly) and N-acetylcysteine (NAC)**, extends lifespan by 24%, enhances mitochondrial function, and supports collagen synthesis and antioxidant defense (Sekhar, 2021; Kumar et al., 2022). **Calcium alpha-ketoglutarate (CaAKG)**, with anti-inflammatory and metabolic benefits, extends female lifespan by 10.5%–16.6%, improving skin hydration and barrier function (Asadi Shahmirzadi et al., 2020; Yang et al., 2022). **Nicotinamide mononucleotide (NMN)**, an NAD^+^ precursor, extends median lifespan by 8.5%, delays frailty, protects against mitochondrial dysfunction, and supports keratinocyte defense against oxidative damage and inflammation (Brito et al., 2022; Kane et al., 2024; Zhang et al., 2024).

These natural geroprotectors target key aging pathways, extending lifespan and supporting longevity-focused cosmeceuticals. Validating their effects requires biomarker analysis, skinspan extension studies, and clinical trials. It should be noted that while most of these compounds are naturally derived, NAC is a synthetic molecule, and for industrial applications, naturally occurring compounds such as NMN and CaAKG are often produced through synthetic methods to ensure purity, stability, and scalability.

### 1.3 Testing reversal of aging hallmarks

To assess the reversal of skin aging hallmarks, reconstructed 3D skin and *ex vivo* human skin models offer superior structural and functional replication compared to traditional 2D cultures. These models better predict clinical outcomes, including inflammatory responses, cytokine production, and epidermal lipid profiles (Netzlaff et al., 2005; Eberlin et al., 2020; Klinngam et al., 2022; Wang E. H. C. et al., 2023; Klinngam et al., 2024). Unlike 2D cultures, which lack complexity and are highly sensitive to elevated ingredient concentrations and cosmetic formulations (Badr-Eldin et al., 2022), 3D and *ex vivo* models provide a reliable platform for evaluating longevity interventions (Liu et al., 2022; Ansaf et al., 2023; Lombardi et al., 2024). To measure aging hallmark reversal, we recommend evaluating key biomarkers alongside complementary markers for supporting evidence. Protein-level detection is prioritized due to its direct functional relevance over mRNA levels.

Genomic instability is assessed using DNA damage markers, oxidative DNA damage indicators, and DNA damage response factors. Telomere attrition is evaluated through telomere length measurements, telomerase activity, and telomere maintenance factors. Epigenetic alterations are characterized by DNA methylation clocks, histone modifications, and chromatin regulators. Loss of proteostasis is examined through protein degradation markers, molecular chaperones, misfolded protein aggregates, and proteasome activity, while disabled macroautophagy is assessed separately through autophagy-related proteins and lysosomal activity markers (López-Otín et al., 2023).

Deregulated nutrient sensing is monitored via metabolic signaling regulators and growth factor signaling activity, while mitochondrial dysfunction is identified through mitochondrial membrane potential assays, oxidative stress markers, mitochondrial DNA copy number, and mitophagy indicators. Cellular senescence is detected using the senescence-associated β-galactosidase (SA-β-gal) assay, cell cycle regulators, nuclear integrity factors, and senolytic indicators. SASP components, which reflect the secretory phenotype of senescent cells, may be classified under cellular senescence or, for clarity, preferably categorized within chronic inflammation, where pro-inflammatory cytokines and SASP-related mediators are used for assessment. Altered intercellular communication is evaluated by examining cell signaling pathways and junction proteins, and stem cell exhaustion is assessed through epidermal and follicular stem cell markers, along with proliferation indicators. Lastly, dysbiosis is characterized by microbiome composition shifts, microbial diversity indices, and bacterial population imbalances (López-Otín et al., 2023). A detailed list of biomarkers, detection methods, and references is provided in Table 1.

**TABLE 1:** Proposed key and complementary biomarkers for testing reversal of skin aging hallmarks.

Aging hallmark	Key biomarkers (methods)	Complementary biomarkers (methods)	References
Genomic Instability	γ-H2AX foci (IF), 8-OHdG (ELISA)	53BP1 foci, Phosphorylated ATM/ATR (Western blot, IF)	Bakkenist and Kastan, 2003; Chen et al., 2020; Punzo et al., 2024
Telomere Attrition	Telomere length (TRF analysis, qPCR, Flow-FISH)	Telomerase activity (TRAP assay), Telomeric Repeat-Containing RNA (TERRA) (qRT-PCR), TRF2 (ELISA, Western blot)	Martinez et al., 2014; Lin et al., 2019; Chang et al., 2020; Jacczak et al., 2021
Epigenetic Alterations	DNA methylation aging clocks (Horvath’s skin and blood clock, epidermis DNA methylation clock)	H3K27me3, SIRT1, TET1 (ELISA, WB)	Kalfalah et al., 2014; Bormann et al., 2016; Horvath et al., 2018; Potic et al., 2020; Wang et al., 2021; Falckenhayn et al., 2023; Kolodziej-Wojnar et al., 2023
Loss of Proteostasis	Total ubiquitinated proteins HSP70/HSP90 (WB, ELISA)	Misfolded protein aggregates (ProteoStat^®^ Staining), Proteasome activity (Fluorogenic Substrate assay)	Imbert et al., 2010; Kleszczyński et al., 2015; Hughes et al., 2022; Cuanalo-Contreras et al., 2023; Stevens et al., 2023; Srivastava et al., 2024
Disabled macroautophagy	LC3-II/I ratio, p62/SQSTM1 (WB, IF)	LAMP1/LAMP2, ATG5, ATG12, ATG7 (WB, IF)	Song et al., 2017; Murase et al., 2020; Arani et al., 2021; Martínez-Martínez et al., 2022; Tsitsipatis et al., 2022; Widgerow et al., 2024
Deregulated Nutrient Sensing	p-mTOR/mTOR ratio, p-AMPK/AMPK ratio (WB, ELISA)	IGF-1/IGFBP ratio (WB, ELISA), FOXO3 (WB)	Yasuoka et al., 2008; Noordam et al., 2013; Ido et al., 2015; Park et al., 2016; Samra et al., 2023
Mitochondrial Dysfunction	Mitochondrial membrane potential (TMRE/JC-1 staining)	ROS levels (DCFDA assay, ELISA), mtDNA/nuclear DNA (qPCR), PINK1/Parkin (WB)	Shen et al., 2015; Crowley et al., 2016; Klinngam et al., 2022; Kelly et al., 2024
Cellular Senescence	SA-β-gal (SA-β-gal assay), p16INK4a (WB, IF)	Lamin B1 (WB, IF), 15-dPGJ2 (ELISA)	Adamus et al., 2013; Zonari et al., 2023
Chronic Inflammation	IL-6, IL-8, TNF-α, GDF15 (ELISA)	MCP-1, GRO-α, MMP-1, MMP-3, CRP (ELISA), COX-2 (WB), PGE-2 (EIA kit)	Lammermann et al., 2017; Heard, 2020; Dorf and Maciejczyk, 2024
Altered Intercellular Communication	Notch1, Jagged1 (WB, IF)	Connexin-43 (Cx43), Wnt3a, β-catenin (WB, IF)	Wan et al., 2021; Yoshioka et al., 2021
Stem Cell Exhaustion	Keratin 15 (K15), p63, LGR5, (WB, IF)	Ki-67, Nestin (WB, IF)	Dos Santos et al., 2015; Dos Santos et al., 2016; Piro et al., 2024
Dysbiosis	Skin microbiome composition (16S rRNA sequencing, shotgun metagenomics), Shannon Diversity Index (qPCR, 16S rRNA sequencing data)	*Cutibacterium acnes* and *Staphylococcus epidermidis* (qPCR)	Carvalho et al., 2023; Forraz et al., 2023; Rikken et al., 2023; Santiago-Rodriguez et al., 2023

8-OHdG: 8-Hydroxy-2′-deoxyguanosine; ATM: ataxia telangiectasia mutated; ATR: Ataxia Telangiectasia and Rad3-Related Protein; ATG: Autophagy-Related Genes; Cx43: Connexin 43; COX-2: Cyclooxygenase-2; CRP: C-Reactive Protein; DCFDA: 2′,7′-Dichlorofluorescein Diacetate; DEJ: Dermal-Epidermal Junction; DNA: deoxyribonucleic acid; ECM: extracellular matrix; EIA: enzyme immunoassay; ELISA: Enzyme-Linked Immunosorbent Assay; GDF15: Growth Differentiation Factor 15; GRO-α: Growth-Regulated Oncogene Alpha; H3K27me3: Trimethylation of Histone H3 at Lysine 27; HSP70/90: Heat Shock Proteins 70 and 90; IF: immunofluorescence; IGF-1/IGFBP: Insulin-Like Growth Factor 1/Insulin-Like Growth Factor Binding Protein; IL-6, IL-8: Interleukin 6 and Interleukin 8; JC-1: 5,5′,6,6′-Tetrachloro-1, 1′,3,3′-tetraethylbenzimidazolylcarbocyanine Iodide; Ki-67: Proliferation Marker Protein Ki-67; LAMP: Lysosome-Associated Membrane Protein; LC3-II/I: Microtubule-Associated Protein 1A/1B-Light Chain 3 Isoforms; MCP-1: Monocyte Chemoattractant Protein-1; MMP: matrix metalloproteinase; mtDNA: Mitochondrial DNA; PGE-2: Prostaglandin E2; qPCR: quantitative polymerase chain reaction; qRT-PCR: Quantitative Reverse Transcription PCR; ROS: reactive oxygen species; SA-β-gal: Senescence-Associated Beta-Galactosidase; SIRT1: Sirtuin 1; SQSTM1/p62: Sequestosome 1; TERRA: Telomeric Repeat-Containing RNA; TET1: Ten-Eleven Translocation Methylcytosine Dioxygenase 1; TNF-α: tumor necrosis factor alpha; TMRE: tetramethylrhodamine, Ethyl Ester; TRAP: telomeric repeat amplification protocol; TRF: terminal restriction fragment; WB: western blot.

### 1.4 Testing “skinspan”: Extending skin viability, structure, and function over time

The term *“skinspan”* is relatively new and has not yet been widely adopted in peer-reviewed dermatology literature. It was first introduced in industry and aesthetic medicine communications, such as the PMFA Journal article by Pearlman (2024), which highlights the importance of maintaining skin integrity over time (Pearlman, 2024). In this context, we define *“skinspan”* as the period of sustained skin viability, structure, and function—measurable through relevant biological and clinical markers. To evaluate the ability of longevity cosmeceuticals to extend “skinspan”—the period of optimal skin health and longevity—we propose assessing skin viability, structure, and function over time using 3D reconstructed or *ex vivo* human skin models.

#### 1.4.1 Assessing skin viability as a reflection of skin lifespan

Skin viability is one of the key determinants of skin lifespan. Measuring skin viability over time provides a functional readout of skin lifespan extension, helping to determine whether longevity cosmeceuticals support sustained skin health and delay overall deterioration.

Viability can be measured through destructive or non-destructive methods. Destructive methods, which require sample processing and prevent further testing, include the MTT assay for mitochondrial activity (Castagnoli et al., 2003; Eberlin et al., 2020), caspase-3/7 activation for detecting apoptosis (Eberlin et al., 2020; Wurbs et al., 2024), Ki-67 staining for proliferation assessment (Wurbs et al., 2024), and live/dead staining, which uses calcein-AM (viable) and propidium iodide (dead) for visual viability analysis (Jin et al., 2022). Additionally, acridine orange staining assesses cellular integrity by fluorescing green in viable cells (double-stranded DNA) and red-orange in single-stranded DNA or RNA, making it a reliable tool for skin viability evaluation (Mohamadali et al., 2023).

In contrast, non-destructive methods allow real-time viability monitoring without tissue termination. The LDH release assay evaluates cytotoxicity based on membrane integrity (Halprin and Ohkawara, 1966; Messager et al., 2003; Bauhammer et al., 2019), the resazurin (Alamar Blue) assay tracks metabolic activity (Garre et al., 2018; Petiti et al., 2024), and the oxygen consumption assay measures mitochondrial activity (Messager et al., 2003; Hong et al., 2020). These methods assess whether active ingredients and formulated products enhance skin viability. For example, a serum containing L-ascorbic acid, ergothioneine, hyaluronic acid, and proteoglycans improved viability in irradiated *ex vivo* skin, with the resazurin assay indicating higher metabolic activity than controls. This suggests protection against solar-induced damage and prolonged skin function (Garre et al., 2018). A summary of methods for assessing skin viability, including both destructive and non-destructive approaches, is provided in Table 2.

**TABLE 2:** Methods for assessing skin viability.

Method type	Assessment target	Assay	References
Destructive methods	Mitochondrial activity	MTT assay	Castagnoli et al., 2003; Eberlin et al., 2020
	Apoptosis	Caspase-3/7 activation	Eberlin et al., 2020; Wurbs et al., 2024
	Cell proliferation	Ki-67 staining	Wurbs et al., 2024
	Cell viability	Live/Dead staining (calcein-AM: viable, PI: dead)	Jin et al., 2022
	Cellular integrity	Acridine Orange staining (dsDNA: green, ssDNA/RNA: red-orange)	Mohamadali et al., 2023
Non-destructive methods	Membrane integrity	LDH release assay	Halprin and Ohkawara, 1966; Messager et al., 2003; Bauhammer et al., 2019
	Metabolic activity	Resazurin (Alamar Blue) assay	Garre et al., 2018; Petiti et al., 2024
	Mitochondrial function	Oxygen consumption assay	Messager et al., 2003; Hong et al., 2020

MTT: 3-(4,5-Dimethylthiazol-2-yl)-2,5-diphenyltetrazolium bromide; PI: propidium iodide; dsDNA: Double-Stranded Deoxyribonucleic Acid; ssDNA: Single-Stranded Deoxyribonucleic Acid; RNA: ribonucleic acid; LDH: lactate dehydrogenase.

#### 1.4.2 Evaluating structural integrity and functional biomarkers

Assessing skin structure and function over time is crucial for evaluating their impact on skin healthspan and lifespan, determining whether interventions can delay structural and functional deterioration. Key aspects include epidermal barrier integrity, dermal-epidermal junction (DEJ) stability, and ECM composition (Eberlin et al., 2020). Key methods for evaluating skin structural integrity and functional biomarkers essential to skin longevity are summarized in Table 3.

**TABLE 3:** Methods for assessing skin structural integrity and function.

Key aspect	Biomarkers (methods)	References
Epidermal Barrier Integrity	-Structural proteins: filaggrin, loricrin, involucrin (IHC/IF) -Tight junctions: claudin-1, occludin, ZO-1 (IHC/IF) -Lipid matrix: ceramides, cholesterol, fatty acids (Lipid chromatography) -Skin barrier function (TEWL)	Rosenthal et al., 1992; Rinnerthaler et al., 2013; Zhang et al., 2014; Brandner et al., 2015; Eberlin et al., 2020; Wang et al., 2020; Kocsis et al., 2022; Yang et al., 2024
Dermal-Epidermal Junction (DEJ) Stability	-Collagen IV, VII, XVII (IHC/IF) -Integrin β4 (IHC/IF) -Laminin-5 (IHC/IF)	Vázquez et al., 1996; Langton et al., 2016; Roig-Rosello and Rousselle, 2020; Jeong et al., 2021; Khalid et al., 2022; Lim et al., 2022
Extracellular Matrix (ECM) Composition	-Collagen I (IHC/IF, Masson’s trichrome staining, ELISA) -Collagen III (IHC/IF, Reticulin staining, ELISA) -Total collagen type I + III (Picrosirius red staining) -Elastin (IHC/IF, ELISA) -Hyaluronic acid (IHC/IF, ELISA) -MMPs (Gelatin zymography, ELISA, IHC) -TIMPs (ELISA, IHC, reverse zymography) -TGF-β (IHC, ELISA)	Lee et al., 1997; Pasquali-Ronchetti and Baccarani-Contri, 1997; Vaalamo et al., 1999; Snoek-van Beurden and Von den Hoff, 2005; Calvi et al., 2012; Quan and Fisher, 2015; Hou et al., 2016; Lee et al., 2016; Šínová et al., 2021; Bhardwaj et al., 2022; Klinngam et al., 2022; Yokota et al., 2022; Zorina et al., 2022; Ivarsson et al., 2023

IHC: immunohistochemistry; IF: immunofluorescence; TEWL: transepidermal water loss; DEJ: Dermal-Epidermal Junction; MMPs: Matrix Metalloproteinases; TIMPs: Tissue Inhibitor of Metalloproteinases; TGF-β: transforming growth factor beta; ELISA: Enzyme-Linked Immunosorbent Assay.

Epidermal barrier integrity protects against water loss, environmental stressors, and microbes, crucial for maintaining skin longevity (Barthe et al., 2024). It consists of keratinocytes, structural proteins (filaggrin, loricrin, involucrin), tight junctions (claudin-1, occludin, ZO-1), and a lipid matrix (ceramides, cholesterol, fatty acids) (Rinnerthaler et al., 2013; Zhang et al., 2014; Brandner et al., 2015; Eberlin et al., 2020; Wang et al., 2020; Yang et al., 2024). Aging depletes these components, accelerating moisture loss and skin aging (Rogers et al., 1996; Feingold, 2009). Restoration of epidermal barrier integrity can be evaluated using IHC/IF, lipid chromatography, and TEWL measurements. (Rosenthal et al., 1992; Brandner et al., 2015; Kocsis et al., 2022).

The DEJ links the epidermis and dermis, facilitating cell adhesion, communication, and renewal (Nishiyama et al., 2000; Roig-Rosello and Rousselle, 2020). Aging and stressors degrade DEJ proteins (collagen IV, VII, XVII, integrin β4, laminin-5), weakening structure, elasticity, and healing (Vázquez et al., 1996; Langton et al., 2016; Khalid et al., 2022). Maintaining DEJ integrity supports skin firmness and regeneration, with IHC and IF used for evaluation (Roig-Rosello and Rousselle, 2020; Jeong et al., 2021; Lim et al., 2022).

ECM composition provides structural support and regulates skin homeostasis (Pfisterer et al., 2021). Key components include collagen I and III for strength (Zorina et al., 2022), elastin for elasticity (Pasquali-Ronchetti and Baccarani-Contri, 1997), and hyaluronic acid for moisture retention (Lee et al., 2016). ECM remodeling, regulated by MMPs, TIMPs, and TGF-β, influences collagen turnover and skin aging (Quan and Fisher, 2015). MMPs serve a dual role: increased expression indicates chronic inflammation and tissue degradation, aligning with the aging hallmark of chronic inflammation, while improved MMP/TIMP balance indicates enhanced ECM remodeling, reflecting improved structural integrity and extended skinspan. To assess ECM integrity, various methods are employed, including IHC, IF, and ELISA to detect collagen type I and III, elastin, hyaluronic acid, MMPs, TIMPs, TGF-β (Vaalamo et al., 1999; Hou et al., 2016; Šínová et al., 2021; Bhardwaj et al., 2022; Sato et al., 2023). Histological stains such as Masson’s trichrome staining for collagen I, reticulin staining for collagen III, and Picrosirius red staining for total collagens I and III provide structural visualization (Calvi et al., 2012). Biochemical assays, including ELISA, allow quantification of collagen I and III, elastin, hyaluronic acid, MMPs, TIMPs, and TGF-β, while gelatin zymography measures MMP activity, and reverse zymography evaluates TIMPs (Lee et al., 1997; Snoek-van Beurden and Von den Hoff, 2005; Lamoral-Theys et al., 2009; Klinngam et al., 2022; Yokota et al., 2022; Ivarsson et al., 2023). These techniques offer comprehensive insights into ECM composition, remodeling, and its role in skin aging.

#### 1.4.3 Advances in long-term skin culture systems

**FIGURE 3:**
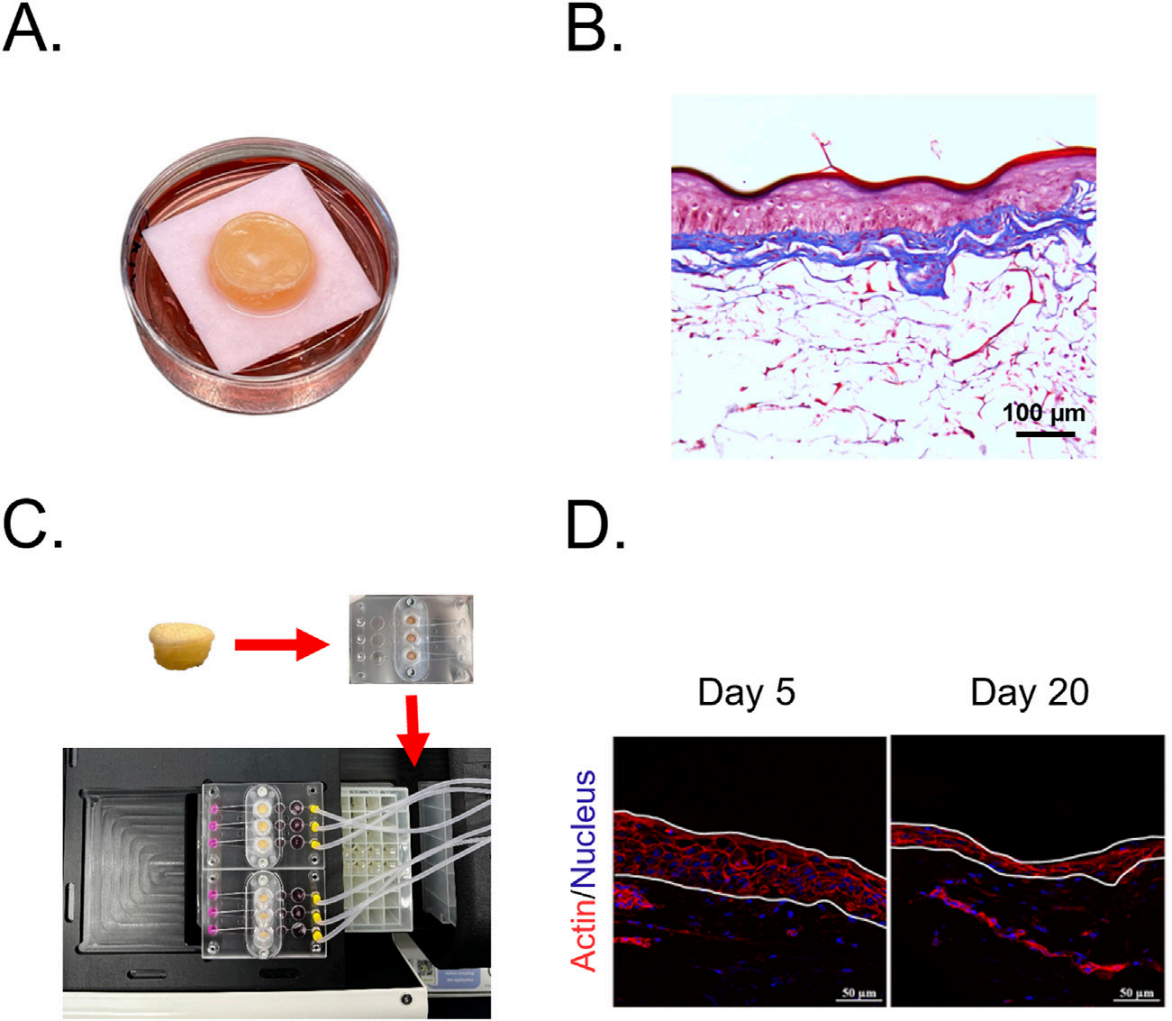
Advanced skin models for long-term culture and aging studies. **(A)** Full-thickness LONG-LIFE 3D Skin, a bioengineered skin model designed for extended viability in culture. **(B)** Histological structure of the full-thickness 3D skin model stained with Masson’s Trichrome, showing a well-developed epidermis and organized dermal matrix. **(C)** Skin-on-a-chip culture system integrating human skin biopsies into a microfluidic platform, enabling long-term perfusion and mechanical stimulation. **(D)** Epidermal thinning in *ex vivo* skin-on-a-chip cultures, visualized through actin (red) and nuclear (blue) staining. Images show a progressive reduction in epidermal thickness from Day 5 to Day 20, a characteristic feature of chronological aging.

Aging biomarkers, such as telomere length and epigenetic clocks, provide critical insights into skin longevity and rejuvenation but require extended treatment periods to observe meaningful changes (Bär and Blasco, 2016; Galow and Peleg, 2022). Traditional *ex vivo* and 3D skin models degrade within 1–2 weeks due to nutrient depletion and metabolic decline, limiting their use in long-term studies (Abaci et al., 2015; Mohamadali et al., 2023; Teertam et al., 2025). To address this, advanced culture systems have been developed, such as Phenion^®^ Full-thickness LONG-LIFE 3D Skin, which extends viability to 50 days (Rimal et al., 2024). As shown in Figures 3A, B (a representative image from our laboratory), this model closely mimics native human skin, featuring epidermal stratification and a well-developed dermal matrix. Its prolonged viability makes it a valuable platform for evaluating skin viability, structure, and function in longevity research. Additionally, microfluidic skin-on-a-chip platforms integrate continuous perfusion, oxygenation, and mechanical stimulation, preserving epidermal and dermal structure for several weeks (Ataç et al., 2013; Abaci et al., 2015; Kim et al., 2019; Jeong et al., 2021; Zoio et al., 2021). As shown in Figures 3C, D (images from our laboratory), prolonged culture in a skin-on-a-chip device gradually thins the epidermis, mirroring features of chronological aging. This highlights its potential as a valuable model for studying long-term skin aging. While further research is needed to optimize these models for assessing key aging markers, particularly epigenetic aging clocks, long-term 3D and *ex vivo* skin cultures will be essential for evaluating longevity cosmeceuticals.

### 1.5 Testing the reversal of skin aging phenotypes through clinical trials

To rigorously confirm the efficacy of longevity cosmeceuticals in reversing skin aging phenotypes, clinical trials should integrate both standard efficacy assessments and biomarker-driven longevity evaluations. While traditional cosmetic clinical tests—such as measurements of skin hydration, elasticity, density, pigmentation, and wrinkle reduction—remain important for evaluating visible improvements, they do not capture the underlying biological mechanisms of skin aging. To substantiate true longevity effects, we propose a longevity-specific assessment framework that complements traditional endpoints with molecular and cellular evaluations. This includes post-treatment skin biopsies and biomarker analyses targeting the hallmarks of aging to determine whether these products genuinely reverse biological skin aging.

Clinical trials evaluating anti-aging cosmeceuticals typically enroll 30 or more subjects, with sample sizes determined by statistical power analysis, expected effect size, and study objectives. Given that visible aging signs (e.g., wrinkles, elasticity loss, uneven pigmentation) become more pronounced after 40, this population is ideal for efficacy assessments (Watson et al., 2009; Sadowski and Sadowski, 2020). Randomized, double-blind, placebo-controlled trials remain the gold standard for minimizing bias and ensuring reliability.

Trials may adopt either a split-face (within-person) or split-group (between-subject) design, each with distinct advantages. Split-face trials minimize inter-individual variability and require smaller sample sizes, making them ideal for topical treatments. However, their validity can be compromised if active treatments induce noticeable side effects (e.g., irritation), potentially biasing subjective assessments. In contrast, split-group trials are necessary for systemic treatments, as they eliminate cross-treatment contamination but require larger sample sizes due to inter-individual differences (Leducq et al., 2023). Careful study design is essential to ensure valid, reproducible outcomes, strengthening the scientific rigor of longevity cosmeceutical evaluations.

#### 1.5.1 Traditional cosmetic clinical tests

Traditional skin assessments are fundamental in dermatological research, enabling the validation of longevity cosmeceutical efficacy. Examples of key parameters include skin hydration, barrier function, surface topography, and mechanical properties (Fischer et al., 2001).

##### 1.5.1.1 Skin hydration and barrier function

Aging leads to progressive hydration loss and weakened barrier integrity, increasing susceptibility to dryness, irritation, and environmental stressors. The stratum corneum, which plays a crucial role in moisture retention and barrier protection, undergoes structural deterioration with age, resulting in reduced hydration levels and increased transepidermal water loss (TEWL) (Wang et al., 2020).

Skin hydration is evaluated using conductance- and capacitance-based methods, which assess water content within the stratum corneum (Wilson et al., 1988). Devices such as the DermaLab Combo, SkiCon, Corneometer, and MoistureMeter SC measure hydration through different principles, including conductance (DermaLab Combo and SkiCon), capacitance (Corneometer), and impedance (MoistureMeter SC) (Kanlayavattanakul and Lourith, 2015a; Akdeniz et al., 2018).

TEWL, a key marker of barrier integrity, reflects water loss from the skin (Wilson et al., 1988). Open-chamber devices (e.g., DermaLab Combo, and Tewameter) provide real-time TEWL measurements but require strict environmental controls (Cohen et al., 2009; Kanlayavattanakul and Lourith, 2015a; Alexander et al., 2018). Closed-chamber devices (e.g., VapoMeter) monitor humidity changes in a sealed environment, allowing for rapid readings, though occlusion effects may impact results (Fluhr and Darlenski, 2014). New ventilated closed-chamber models improve precision by preventing water vapor saturation (Uehara et al., 2023). Given TEWL’s sensitivity to environmental conditions, controlled settings (18°C–21°C, 40%–60% relative humidity) are recommended for consistent measurements (Akdeniz et al., 2018; Alexander et al., 2018).

By providing objective hydration and barrier function data, these assessments are crucial for longevity cosmeceuticals, ensuring formulations enhance moisture retention and skin resilience while supporting long-term skin health.

##### 1.5.1.2 Skin elasticity and firmness

As skin ages, collagen and elastin production decline, leading to reduced elasticity and firmness, which manifests as sagging and wrinkle formation (Muzumdar and Ferenczi, 2021). To objectively assess these changes, instruments such as the Cutometer (Ohshima et al., 2013; Evans et al., 2021) and DermaLab Combo with the skin elasticity probes (Peperkamp et al., 2019; Cheng et al., 2022) are commonly used. These devices utilize negative pressure (suction) to measure skin deformation and recovery, providing insights into viscoelastic properties and collagen-elastin functionality.

Additional tools offer complementary biomechanical assessments: the Reviscometer^®^ RVM 600 analyzes skin viscoelasticity through acoustic wave resonance (Paye et al., 2007). Twistometers, including the Dermal Torque Meter^®^, evaluate torsional resistance to assess elasticity and mechanical stiffness (Kanlayavattanakul and Lourith, 2015b). These biomechanical tools enable quantifiable evaluations of skin elasticity, firmness, and extracellular matrix integrity, making them essential for tracking treatment efficacy in longevity cosmeceuticals.

##### 1.5.1.3 Skin density

Chronological aging leads to reduced skin density, thickness, and collagen organization, affecting tissue stiffness and resilience (Russell-Goldman and Murphy, 2020). High-frequency ultrasound (HFUS) imaging (20–100 MHz) is a widely used non-invasive technique that provides detailed cross-sectional images of the epidermis and dermis, enabling precise thickness and density measurements (Levy et al., 2021; Wang S. et al., 2023). Devices such as the DermaScan C USB and DermaLab Combo (20 MHz probe) visualize dermal density patterns and track collagen organization changes (Peperkamp et al., 2019; Phillips et al., 2020).

Advanced 3D imaging technologies, including ImagePro Opera 3D, Antera 3D, and LifeViz Micro, enable volumetric assessments of specific skin regions, further enhancing dermal structural evaluations (Hurley et al., 2020; Ramirez et al., 2024). These advanced imaging techniques are essential for assessing the efficacy of longevity cosmeceuticals in enhancing skin density, increasing thickness, and restoring ECM integrity.

##### 1.5.1.4 Skin pigmentation and tone

Skin aging is often linked to hyperpigmentation, partly driven by cellular senescence (Kim et al., 2022). Various advanced imaging tools assess age-related pigmentation and treatment efficacy. Narrow-band reflectance meters, such as the Mexameter^®^ MX18, measure skin color by calculating erythema and melanin indices, utilizing wavelengths of 568 nm (melanin absorption), 660 nm (oxyhemoglobin absorption), and 880 nm (hemoglobin absorption) (Clarys et al., 2000; Matias et al., 2015). Chromameters, based on the CIE Lab color space*, quantify skin tone, with increases in lightness (L) and the Individual Typological Angle (ITA°) indicating improvements in skin brightness (Ly et al., 2020).

Additionally, high-resolution imaging systems, including Canfield VISIA and Antera 3D, provide multispectral and topographical analyses, enabling precise tracking of pigmentation changes, melanin distribution, and hemoglobin patterns (Linming et al., 2018). These analytical tools are essential for evaluating longevity cosmeceuticals’ ability to regulate melanogenesis and mitigate aging-driven hyperpigmentation.

Beyond its cosmetic significance, emerging evidence suggests that skin pigmentation serves as a key marker of skin aging, reflecting underlying genetic and cellular aging processes. Notably, pigmentation-associated genes such as MC1R, IRF4, and BNC2 exhibit pleiotropic effects, influencing melanin synthesis as well as age-related traits such as solar lentigines, wrinkles, and perceived age (Ng and Chew, 2022). This highlights the need to consider skin tone changes as a fundamental aspect of longevity-focused skincare interventions.

##### 1.5.1.5 Wrinkle depth, surface roughness, and fine-line reduction

Skin aging leads to decreased collagen production, altered elastin network assembly, and impaired dermal repair, ultimately contributing to wrinkle formation (Veroutis et al., 2021). Advanced imaging techniques allow precise assessment of wrinkle patterns and skin texture.

The VISIA system captures multi-spectral images for detailed wrinkle analysis, while PRIMOS utilizes digital fringe projection to generate high-resolution 3D surface maps for quantitative wrinkle measurement (Nguyen et al., 2021). The Antera 3D system enables multi-layer wrinkle analysis with micrometer precision, while the Visioscan VC 98 evaluates surface roughness and smoothness using image analysis algorithms (Dabrowska et al., 2018; Messaraa et al., 2018).

These advanced imaging techniques are essential for quantifying wrinkle depth, tracking fine-line reduction, and objectively evaluating the efficacy of longevity cosmeceuticals in improving skin texture and minimizing visible signs of aging.

##### 1.5.1.6 Expanding clinical endpoints for skin aging assessment

Emerging research highlights that clinical trial endpoints—such as hydration, elasticity, and pigmentation—capture only a limited portion of the broader skin aging process. In fact, commonly studied features like wrinkles, pigmentation, and photo-aging explain only one-fourth of the total variance in skin aging phenotypes (Ng et al., 2025). Additional underrepresented markers, including telangiectasia, poor lip fullness, ephelides (freckles), ptosis (droopy eyelids), eyebags, and low eyebrow positioning, contribute another quarter of the variance yet are often overlooked in standard cosmetic evaluations (Ng et al., 2025). Integrating these less commonly measured but significant phenotypic markers into clinical methodologies could improve the accuracy and relevance of longevity cosmeceutical assessments, offering a more comprehensive understanding of aging-related changes and enhancing treatment efficacy evaluation.

#### 1.5.2 Biopsy-driven biomarker analysis for validating longevity effects

Standard clinical assessments track visible signs of skin aging and structural changes, such as skin density, but fail to capture the deeper biological processes at the molecular and cellular levels (Figure 2). To address this limitation, post-trial skin biopsies enable the analysis of aging hallmark biomarkers, particularly epigenetic aging clocks, such as Horvath’s Skin & Blood Clock, offering an objective measure of biological age reduction. By revealing underlying rejuvenation mechanisms, rather than just surface-level improvements, this approach strengthens the scientific validation of longevity cosmeceuticals, distinguishing them from conventional skincare products.

Recent studies highlight the value of biopsy-driven biomarker analysis for evaluating longevity interventions. Naughton et al. (2023) initially demonstrated reduced cellular senescence (H2A.J), improved ECM integrity, and favorable gene expression changes related to loss of proteostasis, stem cell exhaustion, and altered intercellular communication using preclinical models. Subsequently, a 24-week placebo-controlled clinical trial confirmed significant improvements in facial sagging, wrinkles, photodamage, and hyperpigmentation. Post-treatment biopsies validated these biological improvements in cellular senescence, ECM integrity, and epidermal barrier function (Naughton et al., 2023).

Similarly, Falckenhayn et al. evaluated dihydromyricetin (DHM) in a clinical trial of 19 women (aged 50–65) over 8 weeks. Participants applied a DHM-containing formulation, and biopsy analysis revealed upregulation of 34 age-related genes previously silenced by DNA methylation, including 23 linked to wrinkle formation. While epigenetic aging clocks were not assessed in biopsies, the study demonstrated in human keratinocytes that DHM significantly reduced DNA methylation age in human keratinocytes, supporting its potential for epigenetic age reversal (Falckenhayn et al., 2023).

By integrating molecular biomarkers into clinical assessments, biopsy-driven analysis establishes a crucial link between visible improvements and the modulation of aging hallmarks, reinforcing the classification of these products as longevity cosmeceuticals.

### 1.6 Safety evaluation of longevity active ingredients and cosmeceutical products

Ensuring the safety of longevity cosmeceuticals requires a comprehensive approach to meet regulatory standards and protect consumers. For new active ingredients with limited safety data, *in vitro* assays are essential in preclinical evaluations. Skin irritation is assessed using the Reconstructed Human Epidermis (RhE) Test (OECD TG 439), which measures cell viability in epidermal models (OECD, 2010). Phototoxicity tests (e.g., 3T3 NRU test, OECD TG 432) evaluate the risk of a substance causing adverse skin reactions in the presence of UV light (OECD, 2019), while skin sensitization assays (e.g., ARE-Nrf2 luciferase test, OECD TG 442D; h-CLAT, OECD TG 442E) detect potential sensitizers through immune activation markers (OECD, 2024a; OECD, 2024b). Genotoxicity assessments (e.g., Ames test, OECD TG 471; micronucleus test, OECD TG 487) determine a substance’s potential to cause DNA damage or mutations (Azqueta and Dusinska, 2015; OECD, 2020; OECD, 2023).

To assess long-term safety, carcinogenicity studies have traditionally relied on long-term animal testing to detect cancer risks (OECD, 2014). However, modern alternatives, such as Cell Transformation Assays (CTAs), offer a more ethical and efficient option by modeling key stages of carcinogenesis without animal use. These assays, including BALB/c 3T3 and Bhas 42, detect early-stage cellular transformations, providing insights into tumor initiation and promotion, closely mimicking multi-stage carcinogenesis despite being *in vitro* models (Steinberg, 2017).

Clinical safety testing of longevity cosmeceutical actives and products should include at least two assessments for skin irritation and skin sensitization, ensuring thorough evaluation of local and allergic reactions. Single patch tests (24–48 h) are commonly used for skin irritation assessments, evaluating erythema and edema. Additionally, the Cumulative Irritation Test (CIT) is recommended to assess whether repeated application leads to progressive irritation, which is critical for daily-use products (Walters et al., 2015). For skin sensitization testing, the human repeated insult patch test (HRIPT) remains the gold standard, consisting of an induction phase (repeated exposure) followed by a challenge phase to assess allergic responses (Politano and Api, 2008). Beyond these core assessments, additional clinical safety tests should be tailored to the active ingredient and intended product application. Photosensitization and phototoxicity tests are recommended for sun-exposed products to ensure they do not trigger UV-induced reactions (Kerr et al., 2010; Lozzi et al., 2020) Similarly, ocular irritation testing is necessary for eye-area formulations to assess potential irritation risks (Gao and Kanengiser, 2004). Additionally, adverse event monitoring should be systematically conducted throughout clinical trials to document and assess any unexpected reactions or side effects, ensuring comprehensive evaluation of product safety.

Although longevity cosmeceuticals currently lack a specific regulatory category and clear safety guidelines, implementing rigorous safety assessments at both the active ingredient and final formulation levels ensures consumer safety, supports regulatory compliance, and advances the scientific validation of effective longevity interventions.

## 2 Discussion

### 2.1 Proving longevity cosmeceutical actives and products

Longevity cosmeceuticals must demonstrate true biological aging reversal, beginning with evaluations of their effects on biomarkers associated with aging hallmarks through laboratory testing. Unlike systemic aging research, which relies on lifespan assays in animal models, standardized ‘skinspan’ biomarkers for quantifying long-term improvements in skin viability, structure, and function have yet to be clearly established. In this review, we propose a skinspan assessment strategy that utilizes laboratory tests focused on extending skin viability and delaying structural and functional deterioration. Epigenetic aging clocks represent a promising biomarker for quantifying biological age reversal; however, further validation is required to establish their reliability and correlation with functional skin outcomes.

Current clinical tests primarily measure visible or structural skin improvements, inadequately capturing the deeper biological mechanisms required to substantiate longevity benefits. To address this limitation, post-trial skin biopsies can directly demonstrate rejuvenation at molecular, cellular, and tissue levels by analyzing aging hallmark biomarkers, consistent with laboratory-based evaluations (Falckenhayn et al., 2023; Naughton et al., 2023). Additionally, rigorous safety evaluations conducted through both *in vitro* assays and clinical trials remain essential for active ingredients and cosmeceutical products (Council, 2009; Barthe et al., 2021). Ultimately, employing these advanced methodologies will clearly differentiate scientifically validated longevity cosmeceuticals from conventional cosmetic skincare.

### 2.2 Challenges in formulation and delivery

Many active ingredients frequently encounter formulation challenges, including instability from oxidation, UV exposure, pH variations, and poor water solubility. Achieving sufficient skin penetration and maintaining bioavailability further complicate their effective use (Oliveira et al., 2022). Advanced encapsulation technologies, such as liposomes, nanoemulsions, and polymeric carriers, help stabilize these ingredients and ensure targeted delivery into deeper skin layers (Salvioni et al., 2021). Innovative delivery methods, including microneedles and penetration enhancers, can additionally enhance absorption through the skin barrier (Badran et al., 2009; Hmingthansanga et al., 2022). Furthermore, the predictive assessments and testing of dermal penetration should be performed to confirm that active ingredients reliably reach their cellular targets and exert meaningful biological effects beyond superficial skin layers (Liang et al., 2024).

### 2.3 Navigating regulatory challenges in longevity-focused skincare

Increasing consumer demand for longevity products now extends beyond pharmaceuticals and nutraceuticals, prominently encompassing skincare (Wyles et al., 2024). As skincare transitions from superficial enhancement toward products capable of modulating aging hallmarks and achieving true rejuvenation, clear regulatory guidelines become increasingly essential. Currently, the term “cosmeceutical” lacks official recognition by most regulatory authorities, with products typically classified strictly as either cosmetics or drug (Pandey et al., 2025). Nevertheless, several countries have established intermediate regulatory categories bridging cosmetics and pharmaceuticals, such as “quasi-drugs” in Japan, “functional cosmetics” in South Korea, and “herbal cosmeceuticals” in Thailand (Cho et al., 2017; Ferreira et al., 2022; Food and Drug Administration, 2023). These categories permit mild therapeutic claims beyond conventional cosmetics but require additional regulatory approval supported by demonstrated clinical efficacy and safety data (Pandey et al., 2025). Given these existing frameworks, the introduction of a new regulatory category specifically for longevity cosmeceuticals appears unlikely in the near future. However, if scientifically validated through biomarker-driven clinical studies, longevity skincare products could potentially qualify for expanded claims beyond traditional cosmetic standards without being as restrictive as pharmaceuticals. Without clearly defined regulatory criteria, innovation risks being stifled, while increasing consumer demand could result in misleading claims and compromised market credibility. Establishing explicit regulatory guidelines would promote scientific rigor, responsible innovation, and enhance consumer confidence in longevity skincare products. Nonetheless, implementing rigorous scientific protocols may incur substantial costs, potentially limiting access for smaller enterprises. Balancing scientific rigor with feasibility is essential for broad adoption and sustained innovation.

### 2.4 Future directions and industry impact

This manuscript represents the first comprehensive effort to propose detailed scientific criteria and regulatory guidance specifically for longevity cosmeceutical active ingredients and products. Rapid advancements in aging biology and geroscience have significantly expanded our understanding of aging mechanisms, enabling the development of innovative skincare interventions, including natural geroprotectors already suitable for cosmetic use. Given the rising consumer interest in scientifically validated longevity skincare, clearly defining and validating this emerging product category is increasingly critical.

Unlike traditional cosmetics, longevity cosmeceuticals aim to modulate fundamental biological processes underlying skin aging, delivering measurable, sustained improvements rather than immediate superficial effects. To credibly support these advanced claims, comprehensive evaluation using validated biomarkers of aging hallmarks in laboratory studies and clinical trials will be essential (Wyles et al., 2024). Transparent consumer communication strategies must also be adopted to prevent false or misleading longevity claims, thereby protecting market credibility and consumer trust. Additionally, achieving global harmonization in product definitions and regulatory guidelines will ensure consistent quality standards internationally. Success in this endeavor will depend on active interdisciplinary collaboration among geroscientists, dermatologists, cosmetic formulators, and regulatory experts, facilitating the translation of cutting-edge research into effective, consumer-accessible skincare innovations.

While acknowledging that regulators do not yet formally recognize longevity cosmeceuticals as a distinct product category, the foundational scientific framework provided by this manuscript will serve as a valuable resource for researchers, innovators, industry stakeholders, and policymakers. Ultimately, the proposed guidelines represent an essential blueprint, establishing longevity cosmeceuticals as a scientifically rigorous skincare category that promotes sustained skin health and drives economic growth within the beauty and health industries.
